# Understanding the role of mental pain in suicidal individuals: from clinical to neuroimaging perspective.

**DOI:** 10.1192/j.eurpsy.2024.1647

**Published:** 2024-08-27

**Authors:** M. Pompili

**Affiliations:** Dept. of Neurosciences, Mental Health and Sensory Organs, Sapienza University of Rome, Rome, Italy

## Abstract

**Introduction:**

In the attempt to shed light on the phenomenology of suicide, this contribution focuses on the role of mental pain as a main ingredient of suicide.

**Objectives:**

Previous studies have shown that mental pain, childhood negative experiences, and maltreatment are associated with suicide risk. Neuroimaging studies demonstrated that such emotional pain shares the same neuroanatomical circuit of somatic pain. Furthermore, concepts related to death, failure, or other unfortunate circumstances activate specific cerebral areas in a suicidal individual compared to a non-suicidal subject.

**Methods:**

The author, through a multicenter investigation, conducted a sizeable clinical study on mental pain related to psychiatric disorders and suicide risk. With this aim, a dataset of more than 2200 psychiatric patients is explored to investigate suicide risk, mental pain, childhood trauma, and the role of depressive symptomatology. Implications emerging from neuroimaging studies are investigated.

**Results:**

A framework emerges about the role of childhood traumatization in mediating between suicide risk and mental pain; furthermore, when individuals experience high mental pain and high depressive symptomatology, regardless of the diagnoses, they are exposed to higher suicide risk.

**Conclusions:**

Such results are presented in light of neuroimaging studies’ role in identifying how mental pain and brain activation are detected in suicidal individuals. Therefore, this contribution aims to understand better mental pain’s role in clinical practice and research activities.

**Disclosure of Interest:**

None Declared

